# On AI’s role in training professionals in assisted reproductive technology

**DOI:** 10.3389/frai.2026.1856714

**Published:** 2026-09-08

**Authors:** Yingming Zheng, Qijing Wang, Xijing Chen, Li Xu, Xiangrong Xu

**Affiliations:** 1 Department of Reproductive Endocrinology, Key Laboratory of Reproductive Genetics of National Ministry of Education, Women’s Reproductive Health Laboratory of Zhejiang Province, Women’s Hospital, School of Medicine, Zhejiang University, Hangzhou, China; 2 Department of Ultrasound, Women’s Hospital, School of Medicine, Zhejiang University, Hangzhou, China; 3 Education Department, Women’s Hospital, School of Medicine, Zhejiang University, Hangzhou, China

**Keywords:** artificial intelligence, medical education, reproductive medicine, competency-based education, embryology training

## Abstract

The rapid advancement of Assisted Reproductive Technology (ART) demands equally innovative approaches to professional training. Traditional educational models in reproductive medicine are often limited by inconsistent quality, variable clinical exposure, and prolonged learning curves. This paper proposes a comprehensive, AI-enhanced training framework designed to standardize and accelerate the education of clinicians, surgeons, and embryologists. The framework integrates six core domains: adaptive learning for theoretical knowledge, AI-driven simulations for clinical decision-making, virtual reality for surgical and embryology skills, large language model-based interactions for patient communication training, and automated tools for objective competency assessment. By leveraging technologies such as computer vision, machine learning, and immersive simulation, this platform is intended to deliberate practice in a safe, repeatable environment while potentially reducing training costs and ensuring uniform competency standards. It should be emphasized that this framework remains conceptual at this stage; it has not yet been implemented or empirically validated, and the described benefits are prospective rather than demonstrated. Future directions include the integration of digital twin technology and the development of ethical guidelines to support widespread implementation in reproductive medicine education.

## Background

The escalating global prevalence of infertility, driven by factors such as shifting social environments and delayed childbearing, has positioned assisted reproductive technologies (ART) as a critical medical solution (Liang et al., 2025; Feng et al., 2025). This surge in demand has created an urgent and prominent need for highly skilled reproductive medicine specialists who possess not only solid clinical expertise but also the proficiency to perform complex ART procedures. However, training such experts is uniquely challenging due to the deeply interdisciplinary nature of reproductive medicine, which integrates obstetrics, andrology, embryology, genetics, and ethics (Cimadomo et al., 2024). Traditional apprenticeship models, while foundational, are inherently limited by case variability, a scarcity of hands-on training opportunities for sensitive procedures like oocyte retrieval, and a lack of objective tools to quantify skill progression. These pedagogical gaps result in non-standardized teaching models and curricula across different reproductive centers, creating significant entry barriers for trainees who must master diverse ovulation induction protocols, pharmacological nuances, and ultrasound-dependent surgical skills.

Recent artificial intelligence (AI) advances have opened new avenues for medical education, particularly in technical skills training (Tozsin et al., 2024). AI-enhanced virtual reality (VR) simulation has been shown to improve dexterity and accuracy in risk-free environments across surgical disciplines (Nassar et al., 2021), with VR-based training associated with accelerated skill acquisition, reduces errors, and enhances competencies in general surgery, neurosurgery, orthopedics, and vascular surgery (Halman et al., 2025). Trainees using VR models have demonstrated greater career clarity, motivation, and Objective Structured Clinical Examination (OSCE) scores (Gan et al., 2023). AI-powered systems have been reported to improve surgical training efficiency by up to 40% for complex procedures like laparoscopy and neuroendoscopy (Alaker et al., 2016; Baby et al., 2020), and can provide real-time feedback, objective assessment, and personalized tutoring-comparable to or exceeding expert instruction (Fazlollahi et al., 2022). In low- and middle-income countries (LMICs), AI-enhanced video review has been found to assess laparoscopic skills with human-level accuracy, addressing gaps where traditional apprenticeship is impractical (Ryder et al., 2024). Beyond surgery, AI has shown promise in supports theoretical and communication training. ChatGPT-assisted Problem-Based Learning (PBL) has been associated with improved knowledge assessments (Hui et al., 2025), and virtual patient simulations have been shown to enhance nursing communication and self-efficacy (Shorey et al., 2019; Simms, 2025). Collectively, these advances suggest AI’s transformative potential, offering the possibility of scalable, objective, and personalized training across technical and cognitive domains.

In this context, grounded in the urgent need for digital transformation in medical education, this paper systematically explores the technical pathways and application scenarios of AI technologies in reproductive medicine specialists training. It proposes a comprehensive framework for an AI-enhanced training platform designed to revolutionize education in this field, encompassing clinical skills, theoretical knowledge, diagnostic reasoning, and ethical practice.

## Current applications of AI in reproductive medicine training

ART involves several key stages requiring distinct competencies: controlled ovarian hyperstimulation, oocyte retrieval, insemination (conventional or via intracytoplasmic sperm injection [ICSI]), embryo culture, preimplantation genetic testing (when indicated), embryo transfer, and luteal phase support (Zanettoullis et al., 2024). With the rapid development of AI technology, machine learning algorithms have been explored for optimizing the day of trigger to improve IVF outcomes (Hariton et al., 2021), while AI techniques are increasingly applied for embryo and oocyte classification (Manna et al., 2013).

In terms of skills training, AI technologies may effectively address three fundamental challenges in reproductive medicine education: the inaccessibility, invisibility, and irreversibility of clinical procedures. The field involves numerous micro-manipulations (such as ICSI and embryo biopsy) and high-precision surgeries (including oocyte retrieval and embryo transfer) that are difficult to observe directly and offer limited opportunities for hands-on practice under the traditional apprenticeship model. AI-driven simulation systems have the potential to overcome these limitations by recreating clinical scenarios with high fidelity, enabling rehearsal of rare and complex procedures in a completely risk-free environment. As Bourdon and colleagues observed (Bourdon et al., 2023), simulation initially emerged as an innovative educational method in medicine, but with continuous optimization of simulators, including scientific validation tools and digital technologies such as 3D printing, it now serves as a valuable complement to both initial and continuing education curricula, ultimately contributing to improved patient outcomes in reproductive endocrinology and infertility training.

### Oocyte retrieval training

Oocyte retrieval encompasses ultrasound-guided follicular aspiration and subsequent microscopic oocyte identification-a two-stage process that presents distinct learning challenges. Training clinicians and embryologists to efficiently perform oocyte recovery and accurately identify cumulus-oocyte complexes traditionally requires extensive time and is prone to error. Emerging evidence suggests that combining oocyte recovery with robotic systems and microscopy equipped with computational vision and AI tools holds promise for improving this process (Mutlu et al., 2026). Ultrasound-based training on follicles has been shown to enhance both the accuracy and efficiency of transvaginal oocyte retrieval (Abdullah et al., 2023).

### ICSI training

Unlike conventional insemination, ICSI is a demanding, multi-step procedure requiring delicate removal of cumulus cells, meticulous selection of mature oocytes, and intricate micromanipulation to immobilize a single sperm, penetrate the oocyte’s outer layers, and deliver it into the cytoplasm. This complexity makes ICSI significantly more time-consuming and operator-dependent, with outcomes strictly tied to technical proficiency. Virtual simulation platforms now allow trainees to develop these micro-manipulation skills without risking precious gametes. AI-powered image analysis has been shown to accelerate sperm identification in surgical samples from hours to minutes, seamlessly integrating into lab workflows to boost treatment success rates (Goss et al., 2024). Deep convolutional neural network (CNN) models have demonstrated powerful potential in accurately identifying key landmarks on oocytes and cleavage stage embryos, such as ICSI or assisted hatching (AH) (Jiang et al., 2023). Beyond enabling automation, these novel findings may lay essential groundwork for AI-powered training systems that could provide real-time guidance and objective feedback.

### Embryo transfer (ET) simulation

The American Society for Reproductive Medicine (ASRM), in collaboration with the Swiss company VirtaMed, developed a training simulator for embryo transfer and intrauterine insemination in 2015 (Practice Committee of the American Society for Reproductive Medicine et al., 2017; Baker et al., 2022). This system incorporates ultrasound-guided real-time simulation with integrated imaging and operational feedback, significantly improving trainee proficiency before clinical application. Following its introduction at the ASRM annual meeting, the simulator gained widespread acceptance among reproduction endocrinology and infertility (REI) fellows. The ASRM ET Simulator effectively enhanced skills and confidence, particularly for beginners and when training with complex cases. The educational impact of such simulation is substantial. Heitmann and colleagues in Maryland developed an ET simulator that helps trainees build procedural familiarity (Heitmann et al., 2017), and improved the level of self-confidence (Bourdon et al., 2023) REI physicians who underwent simulated embryo transfer training have been reported to demonstrate improved pregnancy rates in their first ten clinical transfers. This training enhanced their procedural proficiency, shortened the learning curve, and contributed to improved post-transfer pregnancy outcomes.

A notable 11-year retrospective study demonstrated that after more than 30 simulated embryo transfer sessions, senior trainees achieved clinical pregnancy and live birth rates comparable to those of attending physicians (Miller et al., 2022). However, exposure remains limited. A cross-sectional study in the United States found that nearly 44% of third-year fellows had performed fewer than ten embryo transfers during training (McQueen et al., 2020). This evidence has prompted calls in Fertility & Sterility to establish minimum competency-based standards for embryo transfer training during REI fellowship, including defined minimum numbers of supervised procedures (Ryan et al., 2020). Data-driven feedback mechanisms exemplified by AI-enhanced simulation may not only improve training efficiency but also provide the technological foundation for establishing standardized competency assessments across institutions.

### Endoscopic surgery training

Reproductive specialists were early adopters of laparoscopic and hysteroscopic techniques, but time constraints and competing demands have led many to reduce surgical practice. AI has shown potential in enhancing the training of various types of by improving quality metrics, enhancing lesion detection, and guiding anatomical landmark recognition (Ho et al., 2026). Simulation for endoscopic procedures has matured considerably, ranging from simple box trainers to sophisticated electronic full-scenario simulators. These systems have become integral to gynecologic and reproductive surgery education. A survey of REI trainees revealed that all respondents expressed interest in performing hysteroscopic surgery, and over 82% wished to participate in laparoscopic procedures in the future (Chase et al., 2020), underscoring the necessity of incorporating endoscopic simulation during REI fellowship.

## A proposed multidimensional framework for AI-enhanced training

Research indicates that generative AI offers educators tools for innovative teaching through intelligent instructional design, personalized learning pathways, and real-time feedback (Jarry Trujillo et al., 2024). This human-AI collaborative teaching model offers multiple pedagogical benefits. For example, AI can assist educators in generating diverse clinical cases and simulated patients, enabling trainees to practice clinical reasoning, diagnosis, treatment planning, and patient communication in a safe environment (Xu et al., 2024).

**Figure 1:**
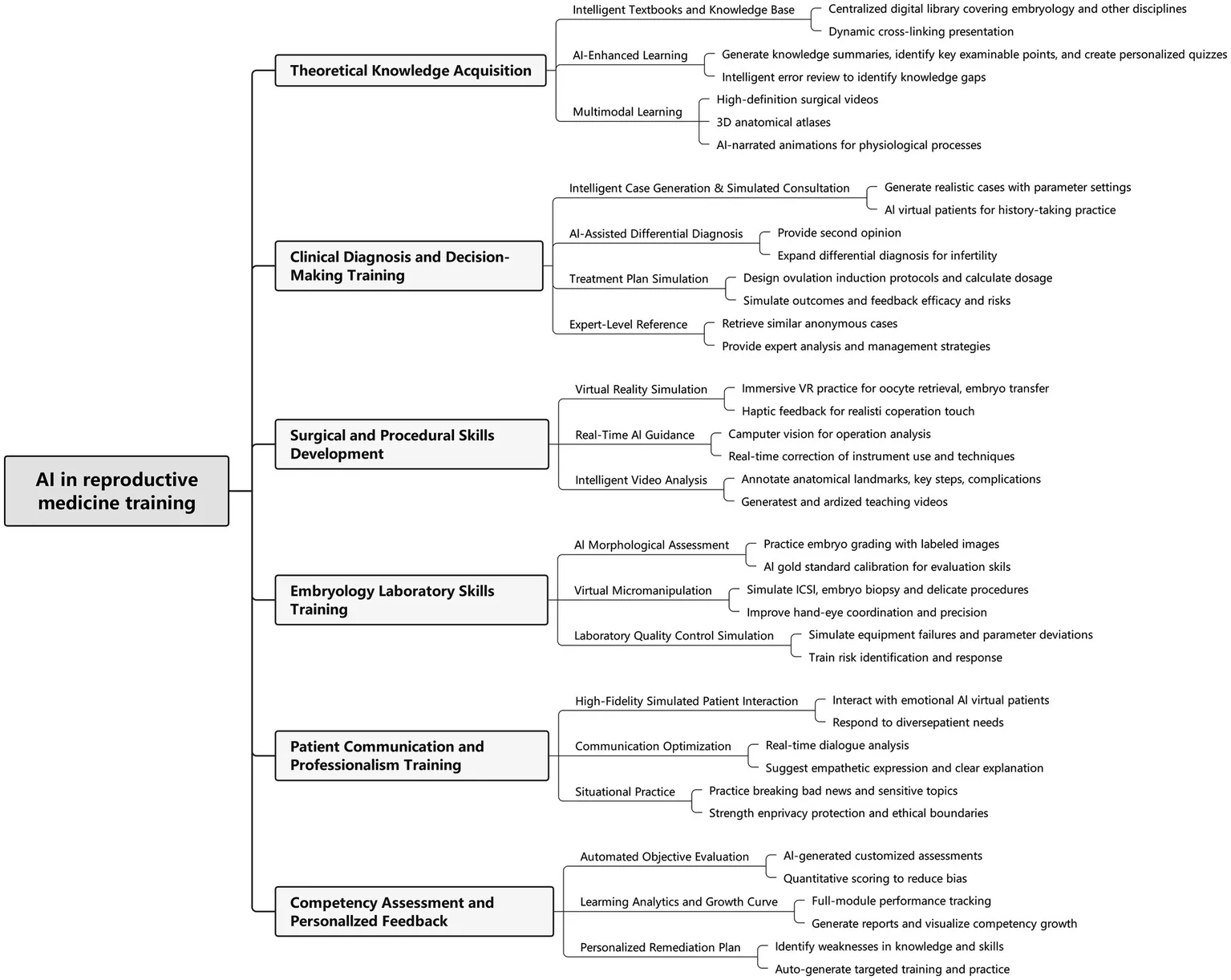
A proposed multidimensional framework for AI-enhanced training.

The proposed platform employs a modular architecture, with each module targeting a critical domain of reproductive medicine training (Figure 1). Their synergy is designed to create a holistic learning ecosystem that addresses a key limitation of existing AI simulation systems-namely, their primary focus on basic operational skills (e.g., embryo placement or oocyte retrieval needle path) with insufficient support for complex competencies such as clinical decision-making, complication management, and doctor-patient communication. Reproductive medicine requires sophisticated cognitive activities-including individualized ovulation induction protocol design and ethical reasoning-where current AI integration remains limited. This platform bridges the gap between technical skill acquisition and advanced competency development.

Technological pillars enabling this framework include computer vision for surgical technique analysis and embryo morphology grading; large language models (LLMs) for intelligent tutoring and patient conversation simulation; VR/AR for immersive skill acquisition; machine learning for personalized learning pathways; and big data with standardized case libraries for rich training corpora. The anticipated impact would include reduced reliance on scarce resources, accelerated competency through deliberate practice, and safe learning environment without patient risk—though these impacts remain prospective, as the framework has not yet been implemented or validated. To better distinguish our proposed integrated ecosystem from currently available AI tools, Table 1 summarizes the key differences in scope, integration, competency coverage, and validation status.

**Table 1:** Comparison of currently available AI applications with the proposed integrated framework.

Dimension	Current AI applications (standalone)	Proposed integrated framework
Scope	Individual tools for specific tasks (e.g., embryo grading, surgical video annotation, virtual patient dialogue)	Comprehensive ecosystem spanning all six competency domains
Integration	Disconnected systems; limited data sharing across tools	Fully integrated with bidirectional module communication and unified progress tracking
Competency coverage	Primarily technical skills (e.g., embryo placement, oocyte retrieval needle path)	Technical + cognitive + communication + ethical competencies
Personalization	Generic feedback or task-specific adjustments	AI-driven personalized learning pathways with adaptive curricula and “booster packs”
Validation	Variable; some tools have published proof-of-concept or single-center data	Framework-level validation pending; phased validation protocol proposed
Deployment	Typically single-modality (e.g., VR-only or quiz-only)	Tiered implementation (full-scale, cloud-based, MVP) adaptable to resource settings

Importantly, the six modules presented in Figure 1 represent a conceptual, integrated educational ecosystem. While each module draws upon published evidence from individual AI technologies or related specialties, the specific combination, and implementation within a unified platform remain hypothetical and untested. The following sections distinguish between external findings—cited to support the pedagogical rationale—and the prospective design of our proposed framework.

### Module 1: theoretical knowledge acquisition

AI breaks down professional silos and promotes interprofessional collaboration through immersive, personalized simulated environments (Guraya, 2024). Moving beyond static textbooks, this module is conceptualized as an intelligent, interactive learning environment via a centralized digital repository covering embryology, andrology, endocrinology, and genetics, with dynamically cross-linked content. LLMs would automatically generate knowledge summaries, identify examinable points, and create personalized quizzes with intelligent error review that pinpoints knowledge gaps. External evidence suggests that 91% of users perceived AI-generated content as more time-efficient than default tools, with high usage frequency demonstrating a significant positive correlation with endorsement of content equivalency (Pearson’s *r*^2^ = 0.55–0.79) (Khurana et al., 2025). The platform is envisioned to integrate multimodal media including high-definition surgical videos, 3D anatomical atlases, and AI-narrated animations explaining physiological processes like folliculogenesis.

### Module 2: clinical diagnosis and decision-making training

Bridging theory and practice in a risk-free environment, this module is designed to generate realistic patient cases by varying parameters such as age, medical history, and diagnostic results. Learners would conduct simulated interviews and practice history-taking with AI-powered virtual patients, then propose diagnoses while the AI offers “second opinion” differential diagnoses. Learners would design ovarian stimulation protocols and calculate medication dosages, with the AI simulating physiological outcomes to provide immediate feedback on efficacy and risks. Supportive evidence includes a reproductive medicine randomized controlled trial (RCT) confirming that AI-assisted case-based learning combined with a flipped classroom significantly improved residents’ theoretical knowledge, clinical decision-making, and Mini-Clinical Evaluation Exercise (Mini-CEX) scores (Wang et al., 2026). And an AI-based ovarian stimulation support system (AACS) achieving area under the curves (AUCs) of 0.964 and 0.948 for ovulation timing and prescription inference (Asada et al., 2024). These findings support the conceptual design of this module, not indicating similar results with the present platform.

### Module 3: surgical and procedural skills development

Leveraging VR and computer vision, this module is conceptualized to create a high-fidelity surgical training environment for procedures such as ultrasound-guided oocyte retrieval and embryo transfer in immersive VR with optional haptic feedback. As learners would perform simulated procedures, computer vision algorithms would analyze movements and provide real-time corrective feedback on instrument handling, technique, and best practices (e.g., flagging excessive manipulation during simulated embryo transfer). The platform is also envisioned to analyze recordings of real surgeries, automatically identifying and annotating key anatomical landmarks, critical steps, and potential complications.

External projects such as GlobalSurgBoxAI (Stanford Medicine) and SOAR (Surgical Objective Assessment and Review) have established the feasibility of applying computer vision to surgical video analysis for performance quantification and feedback provision (Bilbao, 2025; Stanford Surgery, 2025). A prospective study reported that two sessions of high-fidelity simulation training significantly enhanced the efficiency and accuracy of obstetrics and gynecology residents performing oocyte aspiration and embryo transfer while reducing complications (Marin et al., 2025).

### Module 4: embryology laboratory skills training

Embryology is central to reproductive medicine, yet the clinical lab remains a “black box” to trainees, where hands-on experience is constrained by the precious nature of gametes and embryos. Simulation-based education has emerged as a transformative pedagogical strategy (Sarpal et al., 2025), with initiatives such as the ASRM Clinical Embryology Learning Laboratory (CELL) incorporating structured (American Society for Reproductive Medicine, n.d.), hands-on simulation to build foundational competence in a risk-free environment. This module is designed to address this challenge through three complementary training pillars: (a) morphological assessment calibration using an annotated image/video library with AI providing a consensus “gold standard” grade; (b) virtual micro-manipulation simulators for delicate procedures (ICSI, embryo biopsy, vitrification/warming); and (c) simulation of equipment malfunctions and deviations in critical laboratory parameters (temperature, pH) to train environmental risk recognition and response.

### Module 5: patient communication and professionalism training

This module addresses essential soft skills through simulated encounters with emotionally responsive AI avatars representing diverse patient backgrounds and concerns (e.g., a couple anxious about treatment failure, a patient seeking clarification on donor conception) (Karnieli-Miller and Elyoseph, 2026). The AI would analyze learner dialogue in real time, providing suggestions for more empathetic phrasing or clearer explanations of informed consent (Gur et al., 2025). Scenario-based practice would enable navigation of challenging situations (breaking bad news, addressing emotional distress, discussing sensitive topics) with immediate feedback (Chiu et al., 2025). These simulations are intended to reinforce boundaries related to patient privacy and ethical conduct, ensuring comprehensive, patient-centered care alongside technical expertise.

### Module 6: Competency assessment and personalized feedback

Serving as the platform’s central nervous system, this module is conceptualized to track progress and guides future learning through automated assessment. AI would generate customized exams and provides quantitative scores for practical skills based on metrics including accuracy, efficiency, and protocol adherence. The platform would continuously monitor performance across modules, generating analytics and visualizing competency growth over time. By identifying patterns in learner weaknesses, the AI would automatically generate personalized “booster packs” containing targeted training materials, cases, and simulations. A proof-of-concept study demonstrates that AI computer vision can objectively classify surgical skills, potentially assisting human evaluators (Erlich-Feingold et al., 2025); however, further validation is required before routine application.

To ensure synergistic operation of the six modules, the framework incorporates three layers of integration mechanisms: (a) data interfaces enabling bidirectional communication between modules; (b) a learning path recommendation algorithm dynamically generating optimized training sequences; and (c) a unified user progress synchronization system presenting mentors with a comprehensive dashboard for informed oversight.

## Current limitations and challenges

Despite significant progress, several challenges remain in the widespread adoption of AI for reproductive medicine training, including data privacy and security, validation frameworks demonstrating transfer of simulated skills to clinical practice, standardized accreditation pathways, and maintaining the essential role of human mentorship (Elendu et al., 2023; Mohsin Khan et al., 2025).

High-quality training data is scarce, and AI algorithms often lack generalizability (Theodosiou and Read, 2023). Reproductive centers vary widely in imaging equipment, protocols, and annotation standards, and most training systems are developed using single centers data, causing significant performance drops when applied elsewhere. Ideally, datasets should be collected from multiple clinics to represent different populations and conditions, with standardizing imaging protocols across clinics to ensure data consistency (Iannone et al., 2024).

AI-assisted learning also presents potential unintended consequences. A study on nursing students revealed a trade-off between efficiency and competency: while the experimental group saved time, they performed significantly worse in ethical standards and clinical reasoning, attributing this gap to acceptance of AI-generated information without adequate verification (Shin et al., 2024). Similarly, a cohort study on surgical skills training found that AI-enhanced curriculum improved safety performance but negatively impacted efficiency metrics, including reduced dominant hand speed, acceleration, and tumor resection rate (Fazlollahi et al., 2023; Ng et al., 2023). Integrating AI into ART training requires continuous evaluation to ensure transparency and support evidence-based learning objectives.

### Applicability of external evidence to ART training

Throughout this manuscript, we have drawn upon published studies from surgery, urology, obstetrics and gynecology, nursing, and general medical education. However, ART training differs from many other specialties in fundamental ways: the “patient” often comprises a couple or individual with subfertility, introducing dyadic psychosocial dynamics; ART procedures are highly technology-dependent and involve multiple discrete skills distributed across different professionals (clinicians, embryologists, nurses); the margin for error is uniquely constrained by the scarcity of human gametes and embryos; and ART involves profound ethical, legal, and emotional dimensions (e.g., embryo disposition, donor anonymity) that are less prominent in other fields.

These distinctions have important implications. Studies on AI-assisted surgical skills training provide valuable insights into motor skill acquisition but may not fully address the cognitive and communicative complexities unique to ART. Our proposed framework addresses these specialty-specific needs through its modular architecture (explicitly separating clinical, surgical, and embryology domains), ART-specific communication scenarios, and emphasis on team-based training. Nevertheless, much of the cited evidence derives from adjacent fields, and the effectiveness of these approaches in ART training remains to be empirically validated.

### Regulatory and ethical considerations in AI-enhanced reproductive medicine training

The integration of AI into reproductive medicine training raises distinctive regulatory and ethical considerations beyond those encountered in general medical education. These warrant explicit discussion given the unique status of human gametes and embryos, the emotionally charged nature of infertility treatment, and the high-stakes outcomes involved.

Data privacy and patient consent: AI training systems require large datasets—including ultrasound images, surgical videos, and patient clinical histories—for algorithm development and validation. Unlike de-identified data used for research, training data often involves identifiable procedural footage that could potentially compromise patient privacy. Institutions must establish clear protocols for data anonymization, secure storage, and restricted access, with explicit patient consent obtained for use of clinical materials in AI training systems (Elendu et al., 2023). The tiered deployment model proposed here raises additional considerations: cloud-based subscriptions (Tier 2) involve data transmission to external servers, necessitating robust data protection agreements and compliance with local and international regulations (e.g., GDPR, HIPAA).

Algorithmic bias and generalizability: AI models trained on data from predominantly Western, high-resource settings may underperform when applied to diverse patient populations or LMIC contexts. This is particularly concerning in reproductive medicine, where ovarian response, embryo development, and treatment protocols vary significantly across ethnicities, age groups, and geographic regions. Validation studies must therefore include diverse populations to ensure equitable performance across all learner and patient demographics.

Human oversight and professional accountability: While AI can augment training, ultimate responsibility for clinical decisions and procedures rests with human practitioners. Trainees must be explicitly taught to critically appraise AI-generated recommendations rather than passively accept them—a competency that the Shin et al. (2024) study suggests may be eroded by uncritical AI use. The framework’s hybrid model, with AI handling cognitive offloading while human experts provide summative judgments and clinical reasoning guidance, is designed to preserve this critical oversight.

Ethical dimensions unique to reproductive medicine: AI training systems that simulate embryo selection, genetic testing counseling, or donor conception scenarios must be carefully designed to reflect the ethical complexities of these situations. For example, simulations involving preimplantation genetic testing (PGT) should address the ethical tensions between reproductive autonomy and potential discrimination, while donor conception scenarios must incorporate culturally sensitive counseling approaches. The framework’s Module 5 (Patient Communication) is specifically designed to incorporate these ethical dimensions, though the development of ethically sound AI responses will require input from clinical ethicists and patient advocates.

Regulatory pathways and accreditation: Given the current lack of broad consensus on regulatory standards for AI-enhanced training tools in reproductive medicine, some within the field have called for academic bodies to initiate exploratory discussions on their assessment and application—covering aspects such as validation pathways, information transparency, and the role of human review. The validation framework outlined in this work might offer one possible starting point for such conversations.

## Future developments

Future developments are likely to focus on creating fully integrated training platforms that seamlessly combine theoretical learning, procedural simulation, competency assessment, and personalized feedback. Digital twin technology, creating patient-specific virtual replicas for procedure rehearsal, holds particular promise for reproductive surgery training. A randomized trial demonstrated that personalized expert feedback significantly improved surgical performance and skill transfer compared to AI-only instruction, underscoring the critical role of human expertise in AI-assisted training (Giglio et al., 2025). The future of simulation-based learning hinges on a synergistic human-AI partnership: AI would handle cognitive offloading—real-time technical feedback, performance tracking, and documentation—while human experts focus on advanced clinical reasoning, crisis management, ethical counseling, and summative competency judgments. Workflow simulations indicate such automation could reduce mentors’ administrative burden by 50–60%. Crucially, a fail-safe escalation mechanism would ensure persistent deficiencies or outlier errors detected by AI are flagged for immediate human review. These projected benefits remain speculative, derived from modeling and evidence in related fields, and require prospective implementation and rigorous evaluation.

In support of the cost-effectiveness rationale, evidence from the literature demonstrates that simulation training can significantly reduce time to clinical competence, with a transfer effectiveness ratio (TER) of approximately 0.66 (Jiang et al., 2025). AI-guided training has been shown to reduce expert teaching time from hours to approximately 30 min per trainee, with national-level cost savings estimated in the millions of dollars (Driver et al., 2024). Real-world data from the U. S. Veterans Affairs healthcare system confirm that AI-powered VR training reduced per-learner costs by over 90% (from $130 to $11.29) while improving knowledge scores by 12.7% (Kenyon, 2026). Low-cost simulation models using locally sourced materials can be fabricated at less than $1.20 per unit (Wittenberg et al., 2025), and consumer-grade VR headsets ($399–$1,000 per unit) further support a tiered approach (Healio, 2026; Akinwale et al., 2024). Tier 1 figures (~$250K–500K upfront) are informed by published pricing for comparable high-fidelity VR simulators, such as the EyeSi ophthalmic surgical simulator, one of the most established microsurgical VR training systems, which is priced at approximately $250,000–$280,000 with per-trainee annual recurring costs around $19,690 (HelpMeSee, 2025; Nguyen and Kang, 2024). These findings suggest that a sliding-scale implementation strategy could achieve cost-effectiveness across diverse resource settings. However, these figures represent order-of-magnitude estimates, not formal cost analyses from an implemented system.

**Figure 2:**
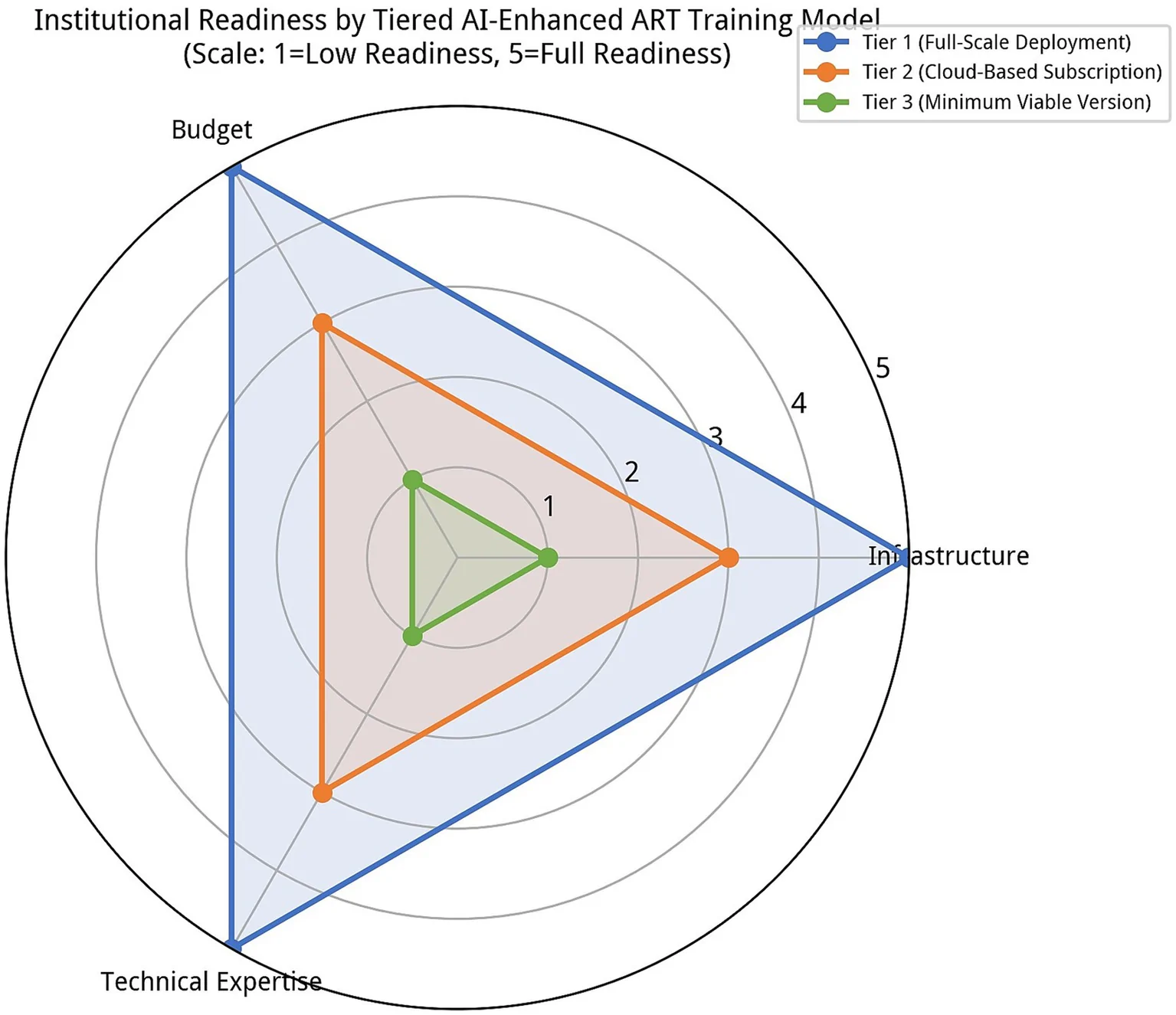
Institutional readiness by tiered AI-enhanced ART training model (Scale: 1 = Low readiness, 5 = Full readiness).

Table 2 summarizes the three deployment options—full-scale, cloud-based subscription, and minimum viable product (MVP)—with Tier 1 (Full-Scale) achieving full readiness (Level 5) for well-funded academic medical centers; Tier 2 (Cloud-Based) attaining moderate readiness (Level 3–4) for regional teaching hospitals; and Tier 3 (MVP) reaching basic readiness (Level 2) for resource-limited settings, with potential for progressive upgrades (Figure 2).

**Table 2:** Tiered implementation strategies for the AI-enhanced training framework.

Resource availability	HIGH 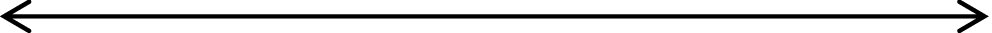 LOW		
Tier	Tier 1 Full-scale deployment	Tier 2 Cloud-based subscription	Tier 3 Minimum viable product (MVP)
Infrastructure	On-premise VR lab High-performance GPUs Haptic devices High-speed network Dedicated IT support	Internet connectivity Cloud compute (AWS/Azure) Subscription license Standard internet speed Vendor support	Basic PC/tablet Web browser Mobile device (offline sync) Low-bandwidth optimization Open-source tools
Modules available	All 6 modules; Full VR immersion Full CV integration Full LLM capacity	Modules 1,2,4,5; Web-based VR (streamed) Cloud LLM API	Module 1 (core); Module 5 (basic OSCE); Basic AI quiz engine Static video library Simplified feedback
Cost	$$$$ (High); ~$250 K–500 K upfront + annual maintenance	$$ (Medium); ~$50 K–100 K annual subscription	$ (Low); ~$5 K–15 K one-time setup + low annual
Target institutions	Academic medical centers Large hospital networks Research institutions	Regional teaching hospitals Mid-size IVF clinics University affiliates	LMIC institutions Small private clinics Resource-limited training programs Remote/rural teaching sites Distributed learning networks
Key advantages	Full immersion Haptic feedback Highest realism All specialties	No capital expenditure Auto-updates Multi-site access Pay-as-you-go	Minimal upfront cost Easy to deploy Highly scalable No IT infrastructure Sustainable in LMICs Low maintenance
Limitations	High upfront cost Space requirements IT maintenance Limited scalability	Internet dependent Latency issues Data privacy concerns Recurring fees	Limited interactivity No haptic feedback Reduced realism Limited module coverage Minimal CV/VR

MVP = minimum viable product; OSCE = objective structured clinical examination; IVF = in vitro fertilization; API = application programming interface; VR = virtual reality; LLM = large language model; CV = computer vision; LMICs = low- and middle-income countries.

Figure 2 illustrates the institutional readiness levels across the three tiered models, rated on a scale from 1 (low readiness) to 5 (full readiness). Tier 1 (Full-Scale Deployment) achieves full readiness (Level 5) with on-premise VR systems, GPUs, haptic devices, and dedicated IT support, suitable for well-funded academic medical centers. Tier 2 (Cloud-Based Subscription) attains moderate readiness (Level 3–4) through cloud VR streaming and subscription-based LLM APIs, designed for regional teaching hospitals with stable internet infrastructure. Tier 3 (Minimum Viable Version) reaches basic readiness (Level 1–2) via a lightweight browser-based implementation with core knowledge and basic assessment modules, tailored for resource-limited LMIC clinics, with the potential for progressive upgrades as funding and infrastructure improve.

## A proposed validation framework for future research

As the proposed platform remains conceptual, establishing a rigorous validation pathway is essential before any claims of educational effectiveness can be made. Grounded in established frameworks for simulation-based training research (Frank et al., 2010; Issenberg et al., 2005; Cook et al., 2011), we outline five phases to guide future implementation.

### Phase 1: pilot implementation and feasibility testing

Developing a minimum viable prototype focusing on one or two modules (e.g., Modules 1 and 2) for deployment in a single academic center. Key outcomes: technical performance (system uptime, latency, usability), institutional readiness, and learner/faculty acceptance via Technology Acceptance Model (TAM) questionnaires. Sample size: 20–30 trainees.

### Phase 2: learner satisfaction and engagement

Mixed-methods evaluation across all six modules with an expanded cohort, combining satisfaction surveys (e.g., System Usability Scale), semi-structured interviews, and focus groups. Attention to cognitive load and simulation authenticity. The MVP would also be tested in an LMIC-affiliated program to assess contextual adaptation.

### Phase 3: competency and educational outcome assessment

Multi-center design (RCT or quasi-experimental with propensity score matching) comparing AI-enhanced vs. conventional training. Outcomes: OSCE scores, procedure-specific metrics (embryo transfer success, oocyte retrieval efficiency) assessed by blinded experts, knowledge pre/post-testing, and Mini-CEX scores. Target: 50–60 trainees per arm based on effect sizes from related studies (Marin et al., 2025; Wang et al., 2026).

### Phase 4: long-term follow-up and transfer of training

Tracking practitioners over 12–24 months on clinical performance indicators (live birth rates, OHSS incidence, complication rates), competency retention, and career progression (time to independent practice, supervisor evaluations).

### Phase 5: comparative effectiveness and cost-effectiveness analysis

Evaluating educational effectiveness, efficiency (time to competency, faculty time), and cost (per-trainee expenditure) from a healthcare educational perspective, incorporating direct and indirect costs. This phase would also assess whether benefits vary across the three tiered models.

We acknowledge that completing all five phases represents a substantial research agenda requiring 3–5 years and multi-institutional collaboration. As an immediate next step, we encourage interested programs to pilot the MVP version with collaborative data sharing. Table 3 summarizes the proposed validation phases with their research questions, methods, outcome measures, and estimated timelines.

**Table 3:** Proposed validation framework for the AI-enhanced training platform: phases, research questions, methods, and outcome measures.

Phase	Research question(s)	Methods	Primary outcome measures
1: Pilot feasibility	Is the system technically feasible and acceptable?	MVP prototype, single-center, *n* = 20–30	System uptime, TAM scores, usability
2: Satisfaction and engagement	Does the platform engage learners without excessive cognitive load?	Mixed-methods, all modules, MVP in LMIC	System usability scale scores, qualitative feedback
3: Competency outcomes	Does the platform improve competency vs. conventional training?	Multi-center quasi-experimental/RCT, *n* = 50-60/arm	OSCE, Mini-CEX, procedure-specific metrics
4: Transfer to practice	Do simulated skills transfer to clinical practice?	Longitudinal follow-up, 12–24 months	Live birth rates, competency retention
5: Cost-effectiveness	Is the platform cost-effective across tiers?	Cost-effectiveness analysis	Per-trainee cost, effect sizes, return on investment

## Conclusion

AI integration into reproductive medicine training marks a paradigm shift toward more effective, equitable, and engaging education. The proposed six-module framework addresses core challenges by making invisible procedures visible, enabling risk-free practice, and delivering data-driven feedback to accelerate skill acquisition. However, this framework remains a conceptual proposal. Throughout this manuscript, we have explicitly distinguished between external findings from published studies—which support the pedagogical rationale—and the prospective functionalities of our integrated platform, which have not yet been implemented or validated. We have also examined the applicability of evidence from adjacent fields to the unique context of ART and highlighted regulatory and ethical considerations specific to reproductive medicine. Realizing this vision requires not only technological development but also rigorous empirical validation through the phased research agenda outlined above. We hope this framework serves as a foundation for future work, ultimately advancing the training of reproductive medicine professionals and improving patient care.

## Data Availability

The original contributions presented in the study are included in the article/supplementary material, further inquiries can be directed to the corresponding author.
